# Lower Within-Community Variance of Negative Density Dependence Increases Forest Diversity

**DOI:** 10.1371/journal.pone.0127260

**Published:** 2015-05-20

**Authors:** António Miranda, Luís M. Carvalho, Francisco Dionisio

**Affiliations:** 1 CE3C - Centre for Ecology, Evolution and Environmental Changes, Faculdade de Ciências, Universidade de Lisboa, 1749–016 Lisboa, Portugal; 2 Plant Biology Department, Faculdade de Ciências da Universidade de Lisboa, Lisboa, Portugal; 3 Instituto Gulbenkian de Ciência, Oeiras, Portugal; Stanford University, UNITED STATES

## Abstract

Local abundance of adult trees impedes growth of conspecific seedlings through host-specific enemies, a mechanism first proposed by Janzen and Connell to explain plant diversity in forests. While several studies suggest the importance of this mechanism, there is still little information of how the variance of negative density dependence (NDD) affects diversity of forest communities. With computer simulations, we analyzed the impact of strength and variance of NDD within tree communities on species diversity. We show that stronger NDD leads to higher species diversity. Furthermore, lower range of strengths of NDD within a community increases species richness and decreases variance of species abundances. Our results show that, beyond the average strength of NDD, the variance of NDD is also crucially important to explain species diversity. This can explain the dissimilarity of biodiversity between tropical and temperate forest: highly diverse forests could have lower NDD variance. This report suggests that natural enemies and the variety of the magnitude of their effects can contribute to the maintenance of biodiversity.

## Introduction

Understanding species co-existence in biotic communities is a fundamental problem in ecological research [1]. How is it possible that so many species co-exist, despite being intensively competing for abiotic factors? This problem is particularly interesting in the case of tropical forests with hundreds of tree species per hectare [2], but the enigma also applies to other types of forests, such as sub-tropical and temperate forests [3], or even to other ecosystems such as coral reefs [4,5].

Arguably, the prominent hypothesis to explain co-existence of species in forests is the one independently proposed by Janzen [6] and Connell [7]. According to their hypothesis, the proximity to adults of the same species reduces seedling survival through attack by host-specific natural enemies, namely insect seed predators and herbivores. Therefore, the probability that a seedling would replace a conspecific dead tree is low. This would give some advantage to other species, preventing competitive exclusion of rarer species. According to this hypothesis, mortality is higher for plant species that occur at higher density, a process called conspecific negative density dependence (NDD). Although initial studies focused more on insect seed predators and herbivores, root pathogens [8] and soil pathogens [9] are now considered to play a major role in the maintenance of forest diversity [10,11]. Indeed these pathogens, rather than insects, mammals and foliar pathogens, seem to cause intense mortality at seed-to-seedling transition [11,12].

Janzen-Connell hypothesis has received strong support [13,14–16]. Nevertheless, it was still unclear how NDD strength variation would influence relative species abundance. The first expectations were that the most abundant species are the ones suffering more from the presence of conspecifics. However, it has been shown that locally rarer species have stronger NDD [3,11,17–19]. Indeed, theoretical studies have shown that, within a forest, there is a positive relationship between NDD strength and relative abundances of tree species [11,20,21]. Surprisingly, Bagchi et al. [12] showed that species with higher seed abundance are the ones suffering more from natural enemies. Despite the discrepancy of these results, all these works show that the variation of NDD within a community have an impact on relative abundances and possibly on the diversity of the community.

One way to access the impact of this NDD strength variation on community diversity, is to compare different communities. Unfortunately, while there are several studies suggesting the importance of the relationship between relative abundance and the interaction between trees and natural enemies within forests, there is still little information about the role of NDD across forest communities. Johnson et al. [3] showed that forests with stronger NDD had higher tree species richness. This relation has been found across a latitudinal study [3], but a recent meta-analysis of several studies showed that the NDD mean value across communities is unrelated with latitude [14]. These studies focus on the comparison of the mean values across forests to explain forest diversity. However, the role of a variation of the NDD range across forests is unclear. To clarify how different ranges of NDD within communities can influence forest diversity, we studied communities: (i) with different mean values of NDD and a fixed variance of NDD; and (ii) with different variances of NDD but the same mean value.

With individual-based computer simulations, we show that stronger NDD leads to higher species diversity. Furthermore, lower variability of strengths of NDD within a community increases species richness and decreases variance of species relative abundances.

## Methods

### Model Description

The model description follows the Overview, Design concepts and Details (ODD) protocol for describing individual based-models proposed by Grimm et al. [22].

#### Purpose

We used a spatially explicit simulation model to investigate the effects of different mean values of NDD strength and different variances of NDD strength; and their impact on community diversity and relative species abundance.

#### Entities, state variables and scales

In order to avoid boundary effects, the simulated landscape is a torus (periodic boundary conditions) composed by a two-dimensional 100 x100 grid and contains two types of entities: grid cells and individuals. Grid cells are characterized by their i- and j-coordinates and can be empty or occupied by one individual. Individuals represent adult trees, and belong to one of 50 species. Each individual is characterized by its location and a species-specific parameter (strength of NDD). All species have a common set of fixed parameters: seed production rate (*f*), adult mortality rate (*m*), mean seed dispersal distance *(d*)—each species differ in the strength of NDD only.

#### Process overview and scheduling

The simulation starts by assigning, to each grid cell, a randomly chosen individual from a set of 50 species. Then, in each time step, a sequence of processes takes place in the following order: adult mortality; seed dispersal; seed mortality; seed establishment (see Table 1 for details).

**Table 1:** Process overview and scheduling.

**Process**	**Pseudo Code**
Adult mortality	For each individual do (die with probability of fixed adult mortality rate).
Seed dispersal	For each empty grid cell do (for each grid cell containing an individual within dispersal distance do (calculate the probability to disperse seeds according to seed production rate and distance from empty grid cell; calculate the number of dispersing seed using binomial distribution and disperse seed to empty grid cell)).
Seed mortality	For each empty grid cell do (for each species do (calculate seed recruitment probability according to the number of conspecific adult trees in 8 the neighboring cells; calculate the final number of seeds in the seed pool according to the seed recruitment probability)).
Seed establishment	For each empty grid cell do (calculate frequency in the seed pool of each species and randomly establish according to frequencies).

#### Design concepts

Species diversity and relative abundances emerge from intraspecific interactions that are a result of the NDD mechanism, which increases the probability of seed mortality with the presence of adult conspecifics. Stochasticity is included in several processes such as initial distribution of individuals in the grid cell; adult mortality; seed dispersal, mortality and establishment. Finally, to evaluate the model output, diversity, relative abundances of all species and NDD strengths of surviving species are observed and registered throughout the whole simulation. The results reported in this study correspond to the outcome of the simulations after 20 000 time steps.

#### Initialisation

All individuals are randomly assigned to a grid cell in the landscape and have equal probability of belonging to one of the 50 species. Therefore, at the start of a simulation each species approximately occupies (100x100)/50 of the grid cells.

#### Input data

The model does not include external data.

#### Submodel adult mortality

Individuals die according to the fixed adult mortality rate (*m* = 0.1).

#### Submodel seed dispersal

We modelled seed dispersal using a negative exponential distribution that is transformed to discrete probabilities (adapted from Banitz et al. [23]):

$$p_{ij}=\;\frac{e^{-\alpha .d_{ij}}}{\sum_{i=-R}^{R}\sum_{j=-R}^{R}e^{-\alpha .d_{ij}}}$$

*i* and *j* are the coordinates of an adult individual with distance $d_{ij}=\sqrt{i^{2}+j^{2}}$ from the focal empty grid cell. The mean seed dispersal distance (*d* = 3, which is fixed for all 50 species) is incorporated by: α=1d and the probability distribution is determined over a total seed dispersal distance of 5 (*R*) grid cells in every direction from the focal empty grid cell. After calculating the discrete dispersal probabilities of the neighboring individuals within the dispersal distance, the actual number of seeds that land in the empty focal grid cell is determined using a binomial distribution. The number of successes is given by the discrete probabilities and the number of trials corresponds to the seed production rate (*f* = 500). At the end of each generation, all the empty grid cells accumulate a seed pool (*N*_*ij*_).

#### Submodel NDD

The density-dependent mechanism is implemented by increasing the probability of seed mortality with the presence of adult conspecifics in the neighboring cells. For example, if NDD = -0.6, then each seed belonging to that species will have a 60% probability of dying. The proportion of surviving seeds of a given species in the focal seed pool decreases with the number of conspecific adults present in the eight nearest cells. For each species, we use four values of NDD corresponding to the following four cases (NDD is stronger when the number of conspecifics increases): 1 or 2 neighbors, 3 or 4 neighbors, 5 or 6 neighbors, and 7 or 8 neighbors. For additional details, see “Relationship between mean value of NDD and species diversity” and “Relationship between variance of NDD and species diversity”.

#### Submodel seed establishment

We calculated the probability of establishment of a species *s* in an empty grid cell by the relative frequency of seeds dispersed by individuals of that species to the grid cell.

$$q_{ijs}=\frac{N_{ijs}}{\sum_{s=1}^{50}N_{ijs}}$$

After calculating all the establishment probabilities, the establishing seed and species is chosen based on its relative frequency (non-biased lottery competition) and a new individual (adult tree) from that species is assigned to the empty grid cell.

### Relationship between mean value of NDD and species diversity

To investigate the impact of different initial NDD strengths with the same NDD variability we generated seven different communities. NDD values of these seven communities span the interval [-1, -0.1] (to assure that all species were suffering with the presence of conspecifics, we chose not to use the value zero). We started with 50 species with the following community mean and range of NDD values: (-0.79, 0.11); (-0.74, 0.11); (-0.68, 0.11); (-0.63, 0.11); (-0.57, 0.11); (-0.51, 0.11); (-0.46, 0.11). The mean community NDD value corresponds to the average of all species NDD values of each community.

In each community, we assured that all 50 species had different NDD strengths by enumerating species from one to 50 and assigning each species NDD values such that the difference of NDD values between two consecutive species was constant. For that, we defined the extremes values of NDD (S1 Table contains NDD values of species one and 50 for each community), and then divided these intervals by 49 to accommodate the 50 species.

**Fig 1:**
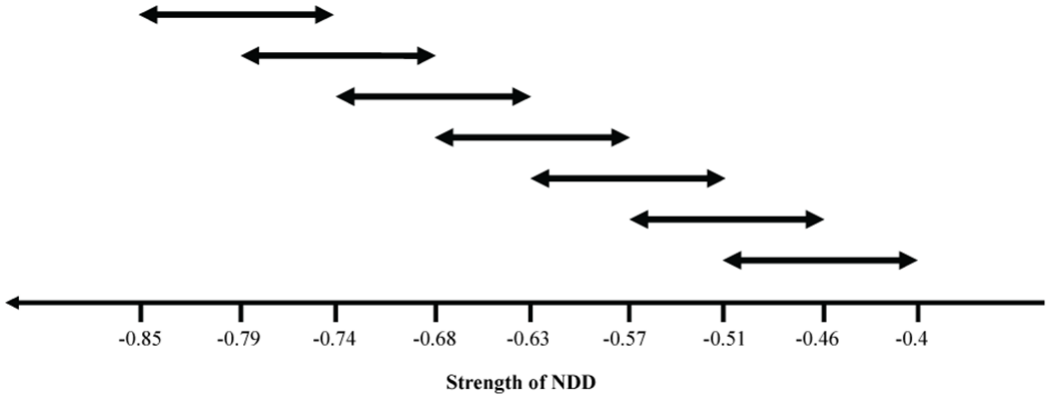
Diagram of the seven initial NDD values with the same initial range but with different initial means of NDD.

There was an overlap between ranges of the communities. This overlap consisted of half the variance being shared with the community with closest lower initial mean values of NDD strength and the other half with the community with the closest higher initial mean values of NDD strength (the two communities in the extremities were an exception for this rule) (Fig 1).

### Relationship between variance of NDD and species diversity

We accessed the influence of different starting NDD strength variances, all with the same initial mean values of NDD strength. We did this by generating ten different communities of 50 species associated with the following community mean and range of NDD values: (-0.63, 0.45); (-0.63, 0.39); (-0.63, 0.34); (-0.63, 0.28); (-0.63, 0.23); (-0.63, 0.17); (-0.63, 0.11); (-0.63, 0.06); (-0.63, 0.03); (-0.63, 0). The mean community NDD value corresponds to the average of all species NDD values of each community.

**Fig 2:**
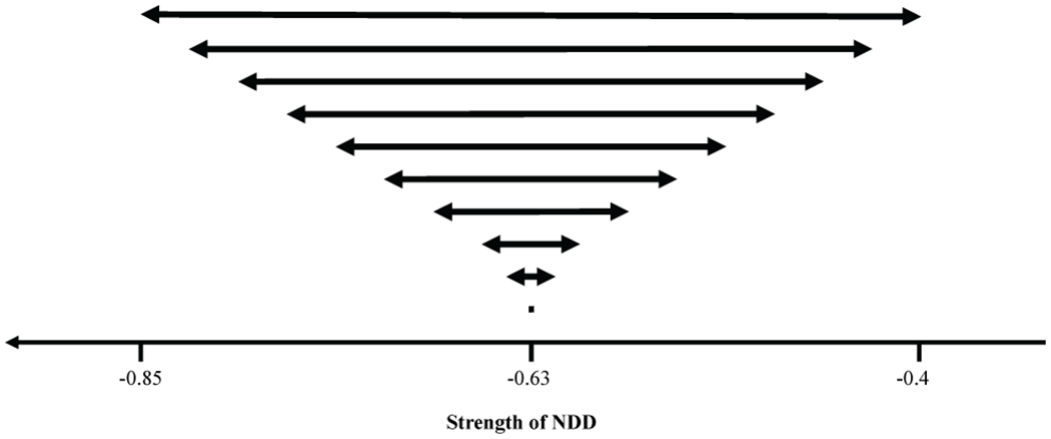
Diagram of the ten initial ranges with different NDD strength variability but with the same initial mean of NDD.

In each community, all 50 species of individuals had different NDD strengths associated. After defining the extremes values of NDD (see S2 Table), we divided these intervals by 49 to accommodate the 50 species. As in the previous simulations, we ensured that the difference of strengths of NDD attributed to two consecutive species is the same within each community. This implies that the difference of NDD between two consecutive species decreases when the range narrows. In the extreme case, we also performed simulations starting with a community with no variance of NDD strength between species (Fig 2).

### Simulation experiments

We used a spatially explicit simulation model that was implemented in MATLAB v8.1 (The Mathworks, Inc.). All simulations started with 10 000 individuals proportionally belonging to 50 different species and stochastically ran through 20 000 generations or 20 000 000 adult deaths. Throughout simulations, the species relative abundance (calculated by dividing the number of adult individuals of a given species by the total number of adult individuals in the grid) and diversity (Shannon index) were registered. All results are based on five community repetitions for each treatment. All statistical analyses were done using IBM-SPSS Statistics v22 (IBM Corporation).

## Results

### Relationship between mean value of NDD and species diversity

**Fig 3:**
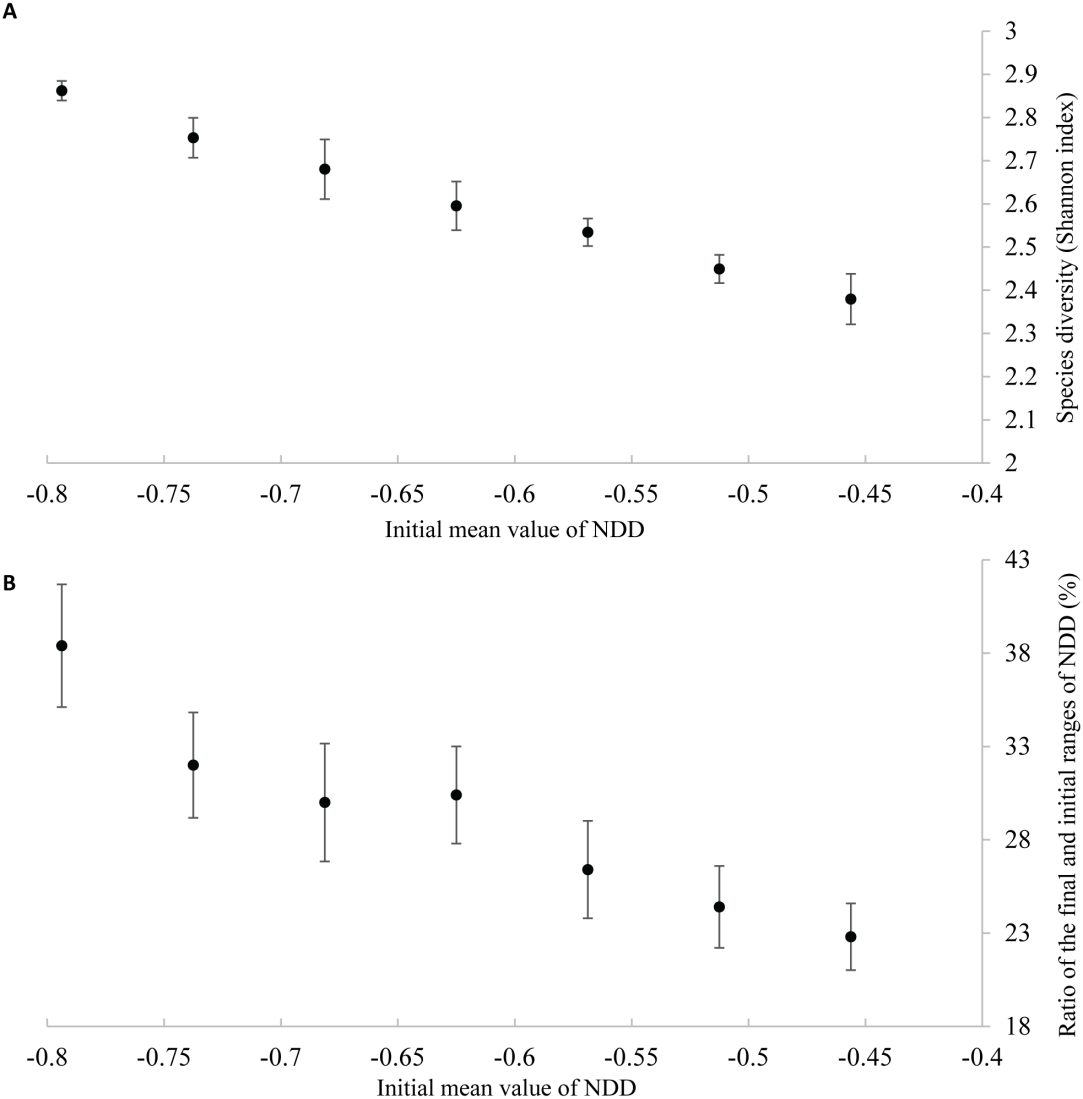
Species diversity (Shannon index) when the initial range of NDD values is fixed and the initial mean of NDD varies (A), and relationship between the initial mean value of NDD and the ratio between final and initial ranges of NDD (B). All results shown obtained at the end of simulations, error bars representing the standard deviation over five repetitions.

We performed simulations beginning with 50 species of trees, each simulation starting with a different initial mean of NDD while maintaining the initial variance, that is, the range of NDD strength at the onset of the simulations (Fig 1). We ran the simulations through 20000 generations because, within this period, most extinctions (at least 94%) occurred within the first 10000 generations (S1 Fig). We found that, at generation 20 000, the number of species (S2 Fig) and the community species diversity (Shannon index) (Fig 3A) was higher for stronger initial means of NDD. We checked the robustness of this conclusion by performing further simulations with other sets of parameters (grid size, number of species, seed production rate, adult mortality rate, and total seed dispersal distance—see S3 Table); we confirmed that species diversity is higher for stronger initial means of NDD (S4 Table and S3 Fig). In Fig 3B, we show that the ratio between the final and initial variances increases with the strength of NDD. Moreover, the final NDD strength is weaker than the initial NDD strength. Therefore, the remaining tree species are the ones with weaker NDD (the species with stronger NDD are extinct), and the stronger is the initial NDD the closer is the final value of NDD to the initial value (regression coefficient = 0.022, *P* = 0.004, S4 Fig).

**Fig 4:**
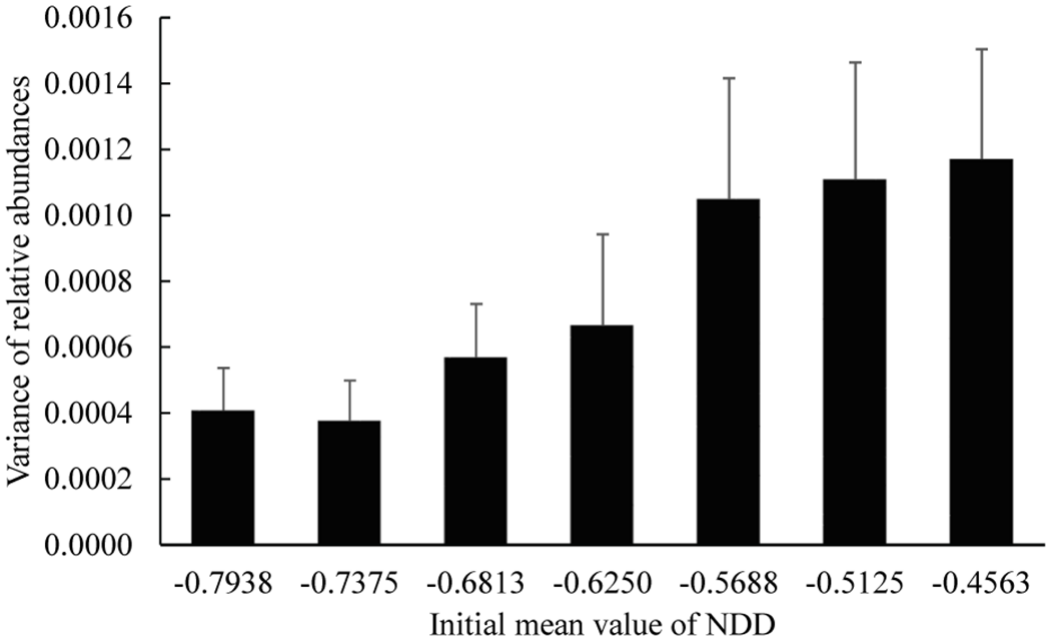
Variance of the relative abundance for different initial mean values of NDD at the end of simulations. Differences of relative abundances between species are lower for stronger initial NDD. Error bars represent the standard deviation over five repetitions.

The mean value of NDD strength has an impact on the relative abundances of each community (S5 Fig): the variance of relative abundances between species is lower for stronger values of NDD (Fig 4).

### Relationship between variance of NDD and species diversity

**Fig 5:**
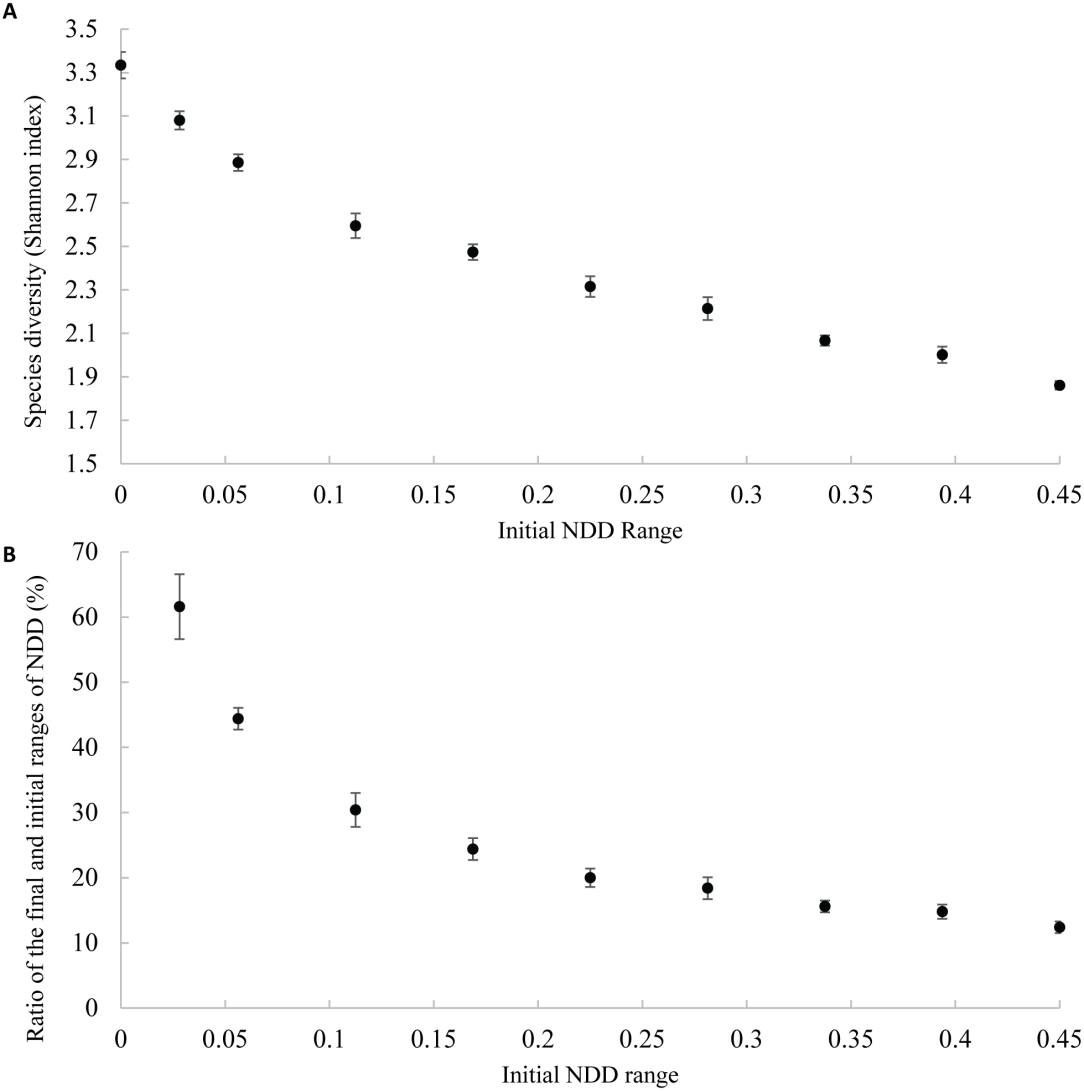
Species diversity (Shannon index) of communities at the end of simulations when the range of NDD values varies between zero (narrower range) and 0.45 (wider range) (A), and relation between initial ranges of NDD and the ratio between final and initial ranges of NDD at the end of simulation (B). Error bars represent the standard deviation over five repetitions.

We performed simulations similar to the previous ones but with ten different initial ranges of NDD strength while maintaining the mean (Fig 2). As before, we ran the simulations through 20 000 generations because, within this period, where there was NDD strength difference between species, most extinctions (at least 89%) occurred within the first 10 000 generations (S6 Fig). We found that, the narrower the initial range of NDD, the higher the number of species at generation 20 000 is (S7 Fig). In particular, we found that forest diversity is maximized when all species have the same value of NDD (Fig 5A). We checked the robustness of the pattern by performing further simulations with other sets of parameters (S3 Table); we confirmed that species diversity is higher for narrower initial ranges of NDD (S5 Table and S8 Fig).

When there is initial NDD variability, the final mean of NDD strength of the remaining tree species is always weaker than the initial strength of NDD (the species with stronger NDD are extinct). Therefore, the final mean value of NDD is closer to the initial mean value of NDD when the initial variance of NDD is lower (regression coefficient = 0.454, *P* < 0.0001, S9 Fig).

Unsurprisingly, in each simulation, the final range of NDD is higher for higher initial ranges. However, the ratio between the final and initial ranges is lower for higher initial ranges (Fig 5B).

**Fig 6:**
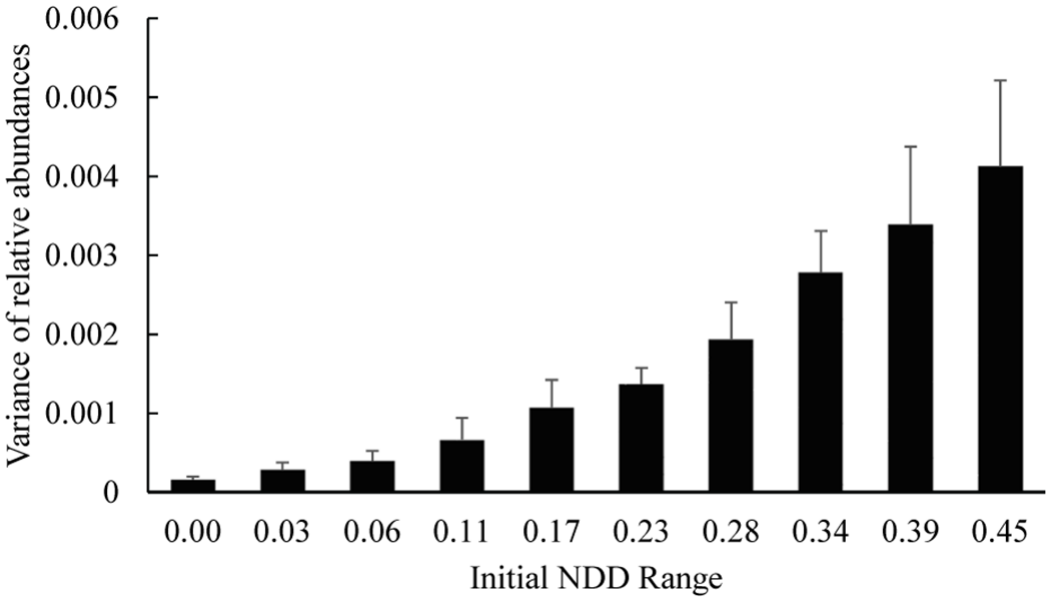
Variance of relative abundances of tree species in different NDD ranges when it varies between zero (narrower range) and 0.45 (wider range) at the end of simulations. Differences of relative abundances between species are higher for wider ranges of NDD. Error bars represent the standard deviation over five repetitions.

Moreover, the species with weaker NDD strength are consistently the most abundant at the end of simulations (S10 Fig). This difference of relative abundances between abundant and rarer species increases with the NDD variance (Fig 6).

## Discussion

We analyzed the role of NDD in structuring forest communities. In particular, we studied the impact of a variability of NDD strength to the plant community structure. For this, we performed two types of simulations: a) we varied the initial mean value of NDD strength (but with the same initial range of NDD values—Fig 1); or b) we initialized the system with initial different ranges of NDD (but with the same initial mean values—Fig 2). We found that the initial values of both the mean and variance of NDD have an impact on the final species diversity and relative abundance.

If the initial NDD range is the same but the mean NDD strength increases (i.e., more negative values of NDD), the number of surviving species increases (Fig 3A and S2 Fig) and the final NDD is always weaker than initially (S4 Fig). These results are in accordance with recent findings that communities with stronger conspecific NDD have higher tree species richness [3]. Moreover, we found that, the stronger the initial NDD, the higher the proportion (ratio between final and initial ranges) of the NDD range is maintained (Fig 3B). Also, by increasing NDD strength, the relative abundance of species with weaker NDD decreases (S5 Fig; see also Fig 4), a relationship also shown by Mack and Bever [21]. Given that the relative abundance of species with weaker NDD decreases, there is more space left for the other species, hence avoiding extinction of some species with stronger NDD.

Interestingly, we show that, the lower the range of initial NDD values is, the higher the final species richness (Fig 5A). There are two main reasons for this effect. First, the wider the NDD range, the higher the competitiveness advantage of the species that are less damaged by the presence of conspecifics. This higher competitiveness implies that their final abundance will be much higher, driving the species that most suffer with the presence of conspecifics to extinction. This implies that the final mean of NDD strength is higher than that of the initial mean (S9 Fig). Second, the narrower the range of NDD is, the more similar the values of NDD associated with tree species (hence lowering the difference of relative competitive advantages). The above arguments also explain why the final variance of relative abundances of tree species is higher for higher initial variances of NDD (Fig 6). Furthermore, there is a relation between the initial and the final NDD range and it is interesting to note that there is a strong drop in the proportion of the NDD variance maintained when the initial variance is low (Fig 5B), showing that a small increase of variance implies the extinction of many species. The expectation was that rare species suffering most with the presence of conspecifics (stronger NDD) are close to extinction, but we show that this extinction is highly dependent on the interaction between overall community NDD mean strength and NDD variance.

Janzen [6] and Connell [7] first hypothesized that seed mortality caused by host-specific plant parasites and predators would be smaller in temperate regions than in tropical regions. Confirming this hypothesis, a recent study using a widespread forest inventory and analysis database of U.S. forests found evidence that the mean regional strength of NDD was correlated with latitude, increasing from boreal to sub-tropical forests [3]. However, in a recent meta-analysis using the results of several experimental studies testing the Janzen-Connell hypothesis, Comita et al. [14] did not find a relation between the strength of density (or distance) dependent mortality and latitude, even when the analysis was restricted to a single region. Therefore, there is still little evidence to support the original hypothesis that the strength of NDD should be higher in the tropics. We suggest another possibility to explain the dissimilarity of biodiversity between tropical and temperate forest, namely the variance of NDD strength within forests and among species. So, highly diverse forests could have lower NDD variance, that is, although the average NDD strength between forests in different latitudes is very similar, the species within temperate forests would have higher NDD differences (as opposed to species in tropical forests with an overall lower variance of NDD strength). Comparative experimental studies between forests in different regions and/or latitude gradients are needed to access the real impact of the total variance of NDD within a forest.

Equally important is to access the causes and/or consequences of the variation of NDD within and among species. Within species there can be variation between different life-stages. While [7] observed NDD at the seedling stage, [6] argued that the NDD effects should exist at both seed and seedling stages. Variation in NDD strengths among species has been linked to physiologically based life history traits, with a positive correlation between shade tolerance and resistance to natural enemies [24]. In our model, in agreement with recent studies [3,11,18] and recent theory [20,21], variations in species relative adult abundance emerge as a consequence of different NDD strength among species in a community. But we also show that NDD variance has a positive relationship with the variance of the species relative abundance (Fig 6). Moreover, there is evidence that, at the seed level, NDD is stronger in more abundant species [12]. This can be due to the fact that seed abundance does not necessarily depend on the tree abundance or can be an indication that species relative abundances can change from seeds to adults, and NDD can play a crucial part in it [12]. Nevertheless, it is still not clear what are the roles of life-stages, species traits, and species relative abundances in driving intra and inter specific NDD variance. In addition, characteristics of parasites such as specificity can be important drivers of the NDD variance [25].

Several theoretical works have already shown that NDD can promote species coexistence [26–28], but our simulations show the important role of NDD variance within and between communities. Using a simulation model, Molofsky et al. [29] has shown that similar spatial patterns may result from multiple mechanisms. Likewise, in this paper, we show that the same final species diversity may result from different initial conditions, namely different initial NDD strengths and different ranges. However, further work, mainly comparative studies measuring the total variance of forest NDD, is necessary to test whether NDD variance is a major player in the maintenance of forest diversity.

## Supporting Information

S1 Fig Percentage of extinctions for seven communities with different initial means of NDD strength. We present the percentage of total extinctions occurred between generations 0–10000 and 10001–20000. Error bars represent the standard deviation over five repetitions.
(DOCX)

S2 Fig Number of species when the initial range of NDD values is fixed and the initial mean of NDD varies. All results shown are obtained at the end of simulations, error bars representing the standard deviation over five repetitions.
(DOCX)

S3 Fig Relationship between mean value of NDD and species diversity, with the parameters shown in S3 Table. In this figure, each letter and associated figure corresponds to the same letter and parameters shown in S3 Table (the set of parameters “e” are the ones used for simulations described in the main text). The initial NDD mean values (horizontal axis) in all figures (a) to (h) are the following: -0.794; -0.625; and -0.456. The vertical axis represent the species diversity (Shannon index). Error bars represent the standard deviation over three repetitions and simulations run for 10 000 generations.
(DOCX)

S4 Fig Relation between initial mean of NDD and distance of the final mean value of NDD to its initial value. Error bars represent the standard deviation over five repetitions.
(DOCX)

S5 Fig Relative abundances for each of the seven communities with different initial means of NDD. From (a) to (g) initial means of NDD are the following: -0.7938; -0.7375; -0.6813; -0.6250; -0.5688; -0.5125; and -0.4563. Error bars represent the standard deviation over five repetitions.
(DOCX)

S6 Fig Percentage of extinctions for ten communities with different initial ranges of NDD strength. We present the percentage of total extinctions occurred between generations 0–10000 and 10001–20000. Error bars represent the standard deviation over five repetitions.
(DOCX)

S7 Fig Number of species of communities at the end of simulations when the range of NDD values varies between zero (narrower range) and 0.45 (wider range). Error bars represent the standard deviation over five repetitions.
(DOCX)

S8 Fig Relationship between variance of NDD and species diversity with the parameters shown in S3 Table. In this figure, each letter and associated figure corresponds to the same letter and parameters shown in S3 Table (the set of parameters “e” are the ones used for simulations described in the main text). The initial NDD ranges (horizontal axis) in all figures (a) to (h) are the following: 0.45; 0.23; and 0.03. The vertical axis represent the species diversity (Shannon index). Error bars represent the standard deviation over three repetitions and simulations run for 10 000 generations.
(DOCX)

S9 Fig Relationship between the initial range of NDD and the distance between initial and final mean values of NDD. (DOCX)

S10 Fig Relative abundances for each of the ten communities with different ranges of NDD. From (a) to (j) ranges are the following: 0.00; 0.03; 0.06; 0.11; 0.17; 0.23; 0.28; 0.34; 0.39.; and 0.45. Error bars represent the standard deviation over five repetitions.
(DOCX)

S1 Table Extreme values of NDD when initial mean values were different but initial range was the same. (DOCX)

S2 Table Extreme values of NDD when initial mean values were the same but differ in the initial range. (DOCX)

S3 Table Parameters used for supplementary simulations. (DOCX)

S4 Table Regression statistics of the relationship between Shannon-index and initial mean values of NDD for different sets of parameters. (DOCX)

S5 Table Regression statistics of the relationship between Shannon-index and initial range of NDD strength for different sets of parameters. (DOCX)
