# Supplementary material for: Lower Within-Community Variance of Negative Density Dependence Increases Forest Diversity
Source: PLoS One. 2015 May 20;10(5):e0127260. doi: 10.1371/journal.pone.0127260 (PMC4439077; doi:10.1371/journal.pone.0127260)
Supplement: S1 Fig — We present the percentage of total extinctions occurred between generations 0–10000 and 10001–20000. Error bars represent the standard deviation over five repetitions. (DOCX) [file pone.0127260.s001.docx]

| 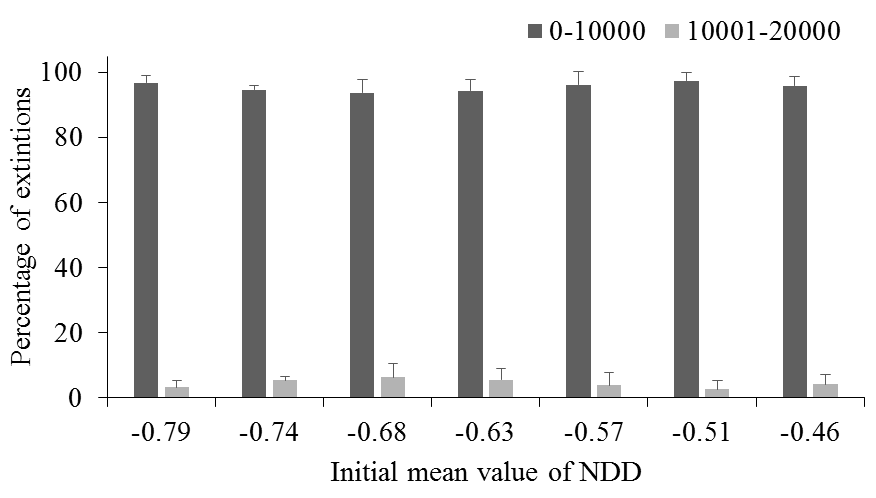 |
| --- |
| S1 Fig: Percentage of extinctions for seven communities with different initial means of NDD strength. We present the percentage of total extinctions occurred between generations 0-10000 and 10001-20000. Error bars represent the standard deviation over five repetitions. |

**Lower within-community variance of negative density dependence increases forest diversity**

António Miranda, Luís M. Carvalho, Francisco Dionisio
