# Supplementary material for: Lower Within-Community Variance of Negative Density Dependence Increases Forest Diversity
Source: PLoS One. 2015 May 20;10(5):e0127260. doi: 10.1371/journal.pone.0127260 (PMC4439077; doi:10.1371/journal.pone.0127260)
Supplement: S2 Fig — All results shown are obtained at the end of simulations, error bars representing the standard deviation over five repetitions. (DOCX) [file pone.0127260.s002.docx]

S2 Fig: Number of species when the initial range of NDD values is fixed and the initial mean of NDD varies. All results shown are obtained at the end of simulations, error bars representing the standard deviation over five repetitions.
