# Supplementary material for: Lower Within-Community Variance of Negative Density Dependence Increases Forest Diversity
Source: PLoS One. 2015 May 20;10(5):e0127260. doi: 10.1371/journal.pone.0127260 (PMC4439077; doi:10.1371/journal.pone.0127260)
Supplement: S3 Fig — In this figure, each letter and associated figure corresponds to the same letter and parameters shown in S3 Table (the set of parameters “e” are the ones used for simulations described in the main text). The initial NDD mean values (horizontal axis) in all figures (a) to (h) are the following: -0.794; -0.625; and -0.456. The vertical axis represent the species diversity (Shannon index). Error bars represent the standard deviation over three repetitions and simulations run for 10 000 generations. (DOCX) [file pone.0127260.s003.docx]

**Lower within-community variance of negative density dependence increases forest diversity**

António Miranda, Luís M. Carvalho, Francisco Dionisio

| **a** | **b** | **c** |
| --- | --- | --- |
|  |  |  |
| **d** | **e** | **f** |
|  |  |  |
| **g** | **h** |  |
|  |  |  |

S3 Fig: Relationship between mean value of NDD and species diversity, with the parameters shown in Table S3. In this figure, each letter and associated figure corresponds to the same letter and parameters shown in Table S3 (the set of parameters “e” are the ones used for simulations described in the main text). The initial NDD mean values (horizontal axis) in all figures (a) to (h) are the following: -0.794; -0.625; and -0.456. The vertical axis represent the species diversity (Shannon index). Error bars represent the standard deviation over three repetitions and simulations run for 10 000 generations.
