# Supplementary material for: Lower Within-Community Variance of Negative Density Dependence Increases Forest Diversity
Source: PLoS One. 2015 May 20;10(5):e0127260. doi: 10.1371/journal.pone.0127260 (PMC4439077; doi:10.1371/journal.pone.0127260)
Supplement: S4 Fig — Error bars represent the standard deviation over five repetitions. (DOCX) [file pone.0127260.s004.docx]

**Lower within-community variance of negative density dependence increases forest diversity**

António Miranda, Luís M. Carvalho, Francisco Dionisio

S4 Fig: Relation between initial mean of NDD and distance of the final mean value of NDD to its initial value.
