# Supplementary material for: Lower Within-Community Variance of Negative Density Dependence Increases Forest Diversity
Source: PLoS One. 2015 May 20;10(5):e0127260. doi: 10.1371/journal.pone.0127260 (PMC4439077; doi:10.1371/journal.pone.0127260)
Supplement: S5 Fig — From (a) to (g) initial means of NDD are the following: -0.7938; -0.7375; -0.6813; -0.6250; -0.5688; -0.5125; and -0.4563. Error bars represent the standard deviation over five repetitions. (DOCX) [file pone.0127260.s005.docx]

**Lower within-community variance of negative density dependence increases forest diversity**

António Miranda, Luís M. Carvalho, Francisco Dionisio

| **a** | **b** |
| --- | --- |
| **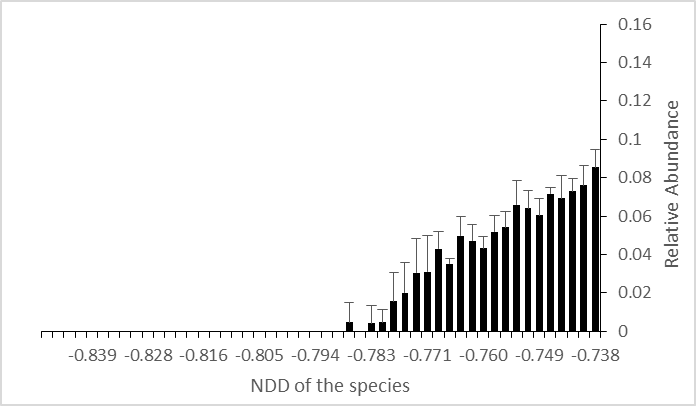** | **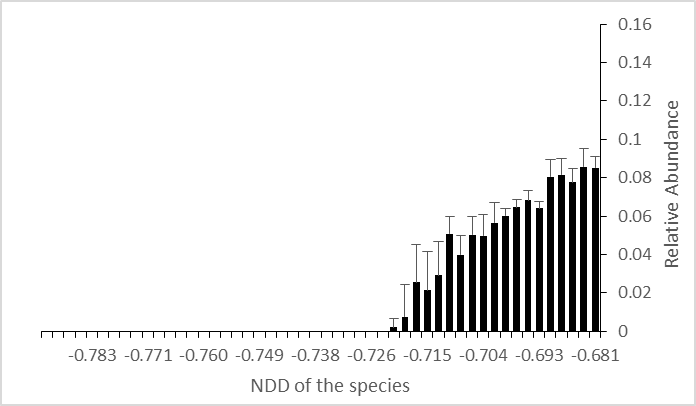** |
| **c** | **d** |
| **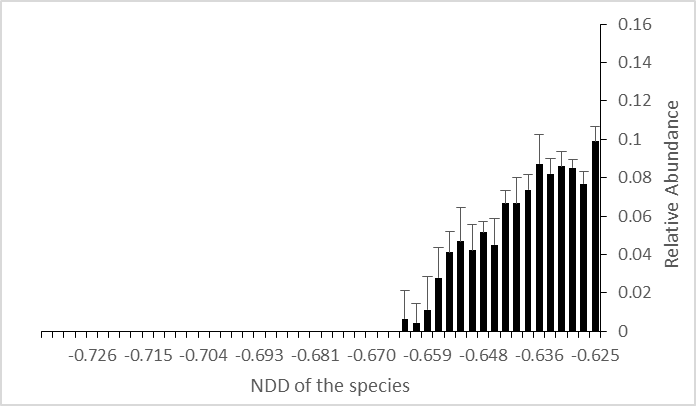** | **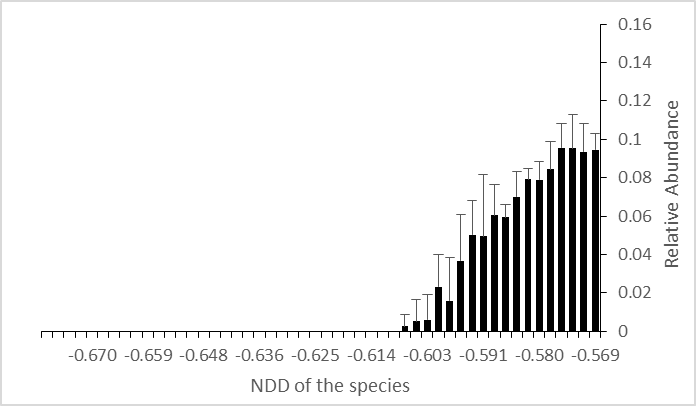** |
| **e** | **f** |
| **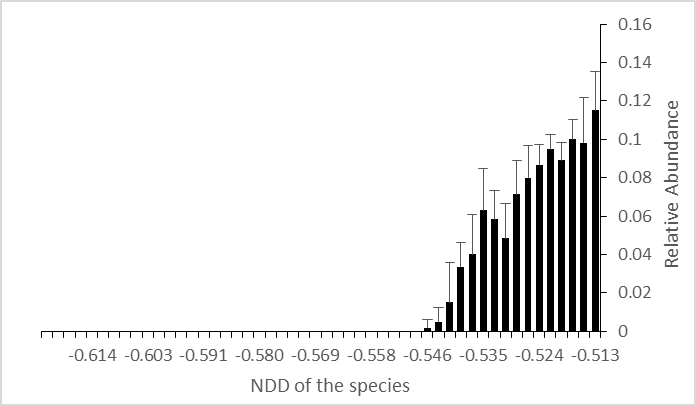** | **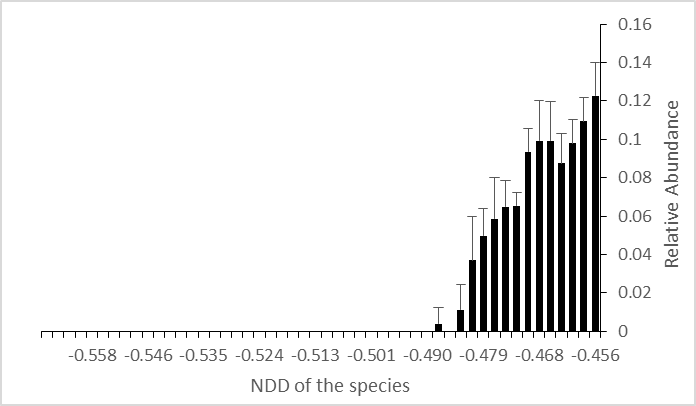** |
|  |  |
| **g** |  |
| **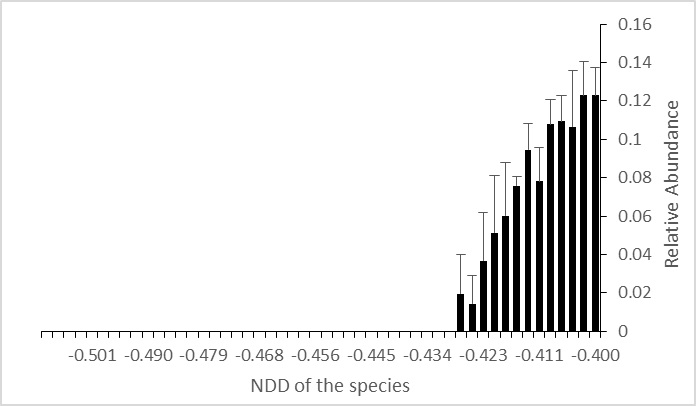** |  |
|  |  |
|  |  |

S5 Fig: Relative abundances for each of the seven communities with different initial means of NDD. From (a) to (g) initial means of NDD are the following: -0.7938; -0.7375; -0.6813; -0.6250; -0.5688; -0.5125; and -0.4563. Error bars represent the standard deviation over five repetitions.
