# Supplementary material for: Lower Within-Community Variance of Negative Density Dependence Increases Forest Diversity
Source: PLoS One. 2015 May 20;10(5):e0127260. doi: 10.1371/journal.pone.0127260 (PMC4439077; doi:10.1371/journal.pone.0127260)
Supplement: S7 Fig — Error bars represent the standard deviation over five repetitions. (DOCX) [file pone.0127260.s007.docx]

**Lower within-community variance of negative density dependence increases forest diversity**

António Miranda, Luís M. Carvalho, Francisco Dionisio

S6 Fig: Number of species of communities at the end of simulations when the range of NDD values varies between zero (narrower range) and 0.45 (wider range). Error bars represent the standard deviation over five repetitions..
