# Supplementary material for: Lower Within-Community Variance of Negative Density Dependence Increases Forest Diversity
Source: PLoS One. 2015 May 20;10(5):e0127260. doi: 10.1371/journal.pone.0127260 (PMC4439077; doi:10.1371/journal.pone.0127260)
Supplement: S10 Fig — From (a) to (j) ranges are the following: 0.00; 0.03; 0.06; 0.11; 0.17; 0.23; 0.28; 0.34; 0.39.; and 0.45. Error bars represent the standard deviation over five repetitions. (DOCX) [file pone.0127260.s010.docx]

| **a** | **b** |
| --- | --- |
| **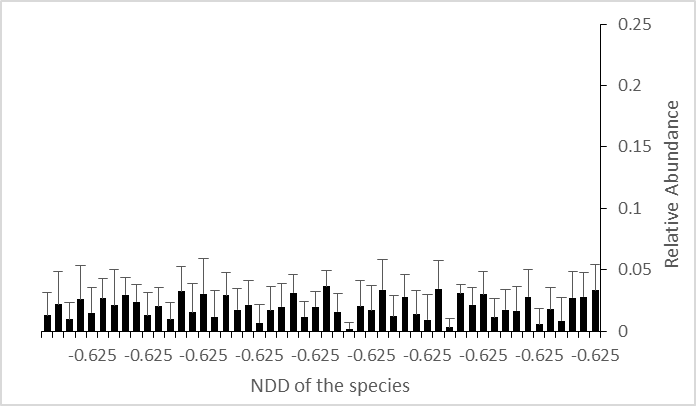** | **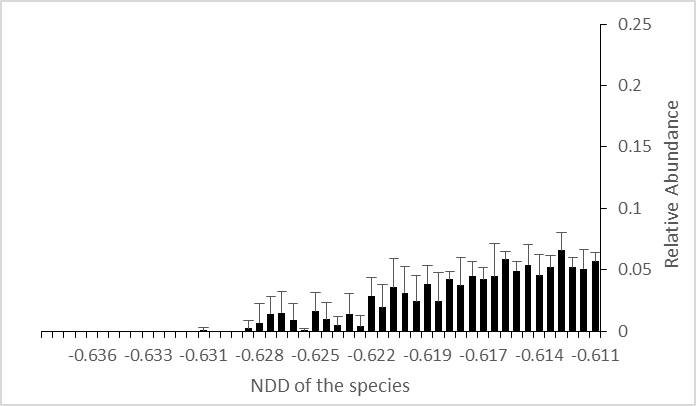** |
| **c** | **d** |
| **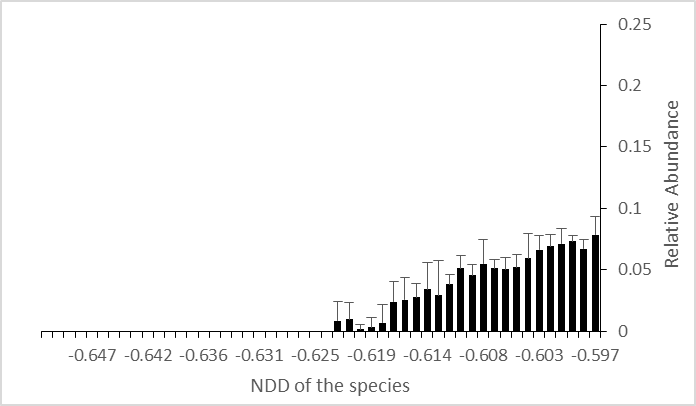** | **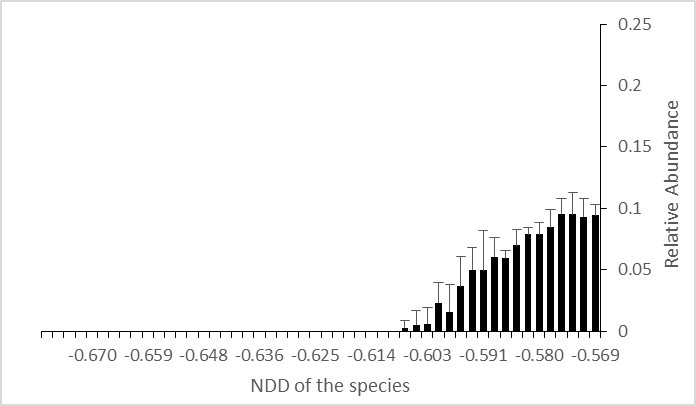** |
| **e** | **f** |
| **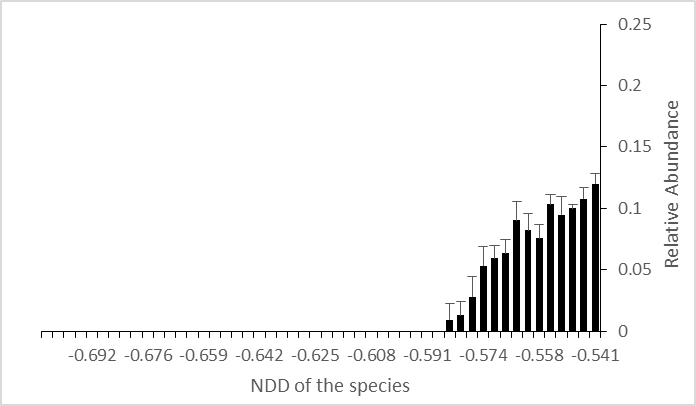** | **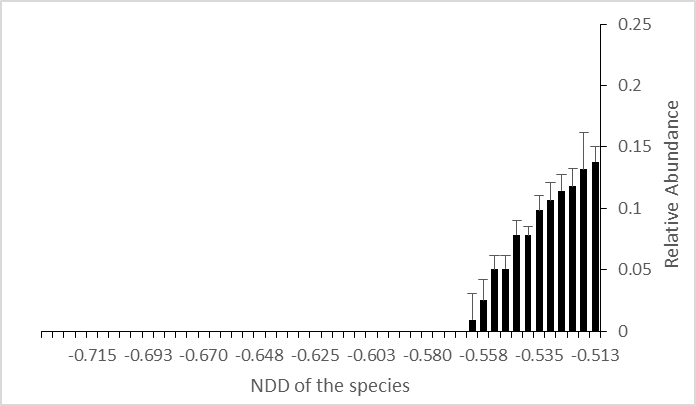** |
|  |  |
| **g** | **h** |
| **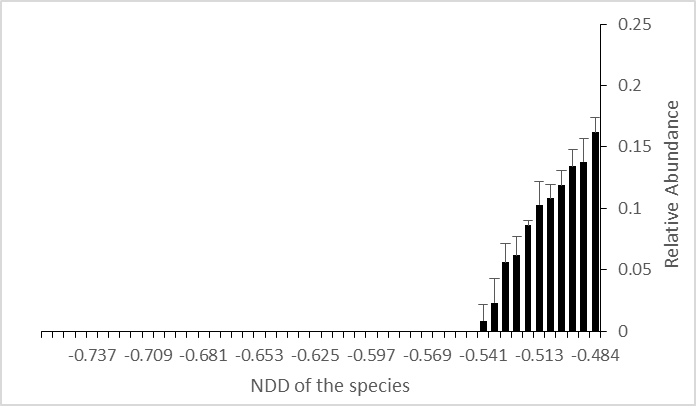** | **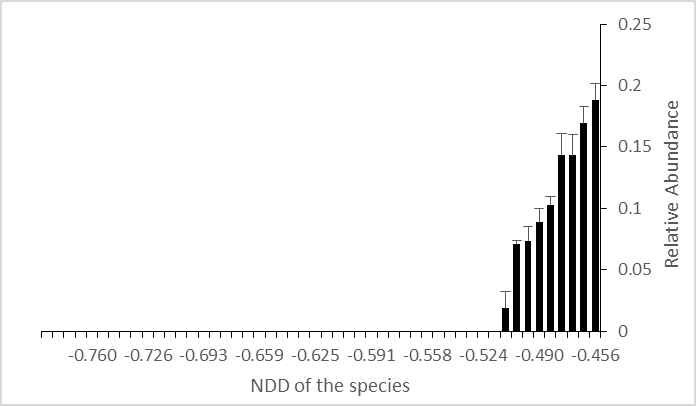** |
| **i** | **j** |
| 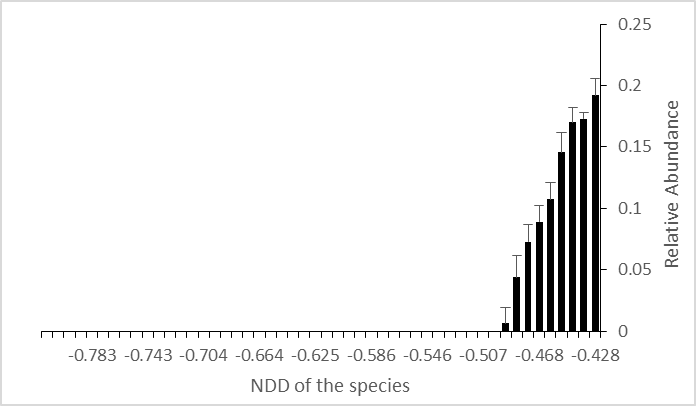 | **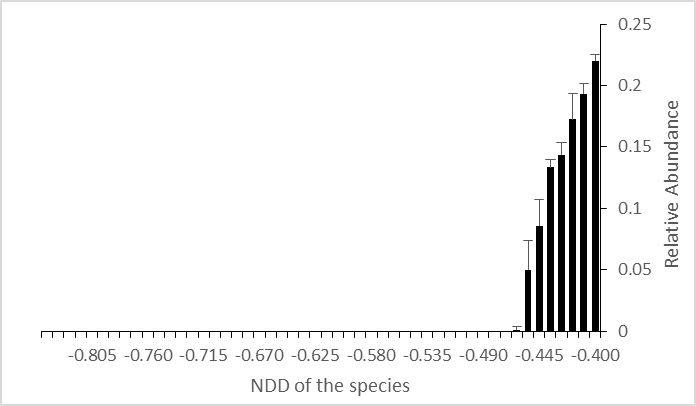** |
|  |  |

S10 Fig: Relative abundances for each of the ten communities with different ranges of NDD. From (a) to (j) ranges are the following: 0.00; 0.03; 0.06; 0.11; 0.17; 0.23; 0.28; 0.34; 0.39.; and 0.45. Error bars represent the standard deviation over five repetitions.
