# Supplementary material for: Lower Within-Community Variance of Negative Density Dependence Increases Forest Diversity
Source: PLoS One. 2015 May 20;10(5):e0127260. doi: 10.1371/journal.pone.0127260 (PMC4439077; doi:10.1371/journal.pone.0127260)
Supplement: S1 Table — (DOCX) [file pone.0127260.s011.docx]

| S1 Table: Extreme values of NDD when initial mean values were different but initial range was the same. | | | | | | | | | |
| --- | --- | --- | --- | --- | --- | --- | --- | --- | --- |
|  | MINIMUM | | | |  | MAXIMUM | | | |
| Nr of conspecific neighbours | 1 or 2 | 3 or 4 | 5 or 6 | 7 or 8 |  | 1 or 2 | 3 or 4 | 5 or 6 | 7 or 8 |
|  | -0.7000 | -0.8000 | -0.9000 | -1.0000 |  | -0.5500 | -0.6750 | -0.8000 | -0.9250 |
|  | -0.6250 | -0.7375 | -0.8500 | -0.9625 |  | -0.4750 | -0.6125 | -0.7500 | -0.8875 |
|  | -0.5500 | -0.6750 | -0.8000 | -0.9250 |  | -0.4000 | -0.5500 | -0.7000 | -0.8500 |
|  | -0.4750 | -0.6125 | -0.7500 | -0.8875 |  | -0.3250 | -0.4875 | -0.6500 | -0.8125 |
|  | -0.4000 | -0.5500 | -0.7000 | -0.8500 |  | -0.2500 | -0.4250 | -0.6000 | -0.7750 |
|  | -0.3250 | -0.4875 | -0.6500 | -0.8125 |  | -0.1750 | -0.3625 | -0.5500 | -0.7375 |
|  | -0.2500 | -0.4250 | -0.6000 | -0.7750 |  | -0.1000 | -0.3000 | -0.5000 | -0.7000 |
