# Supplementary material for: Lower Within-Community Variance of Negative Density Dependence Increases Forest Diversity
Source: PLoS One. 2015 May 20;10(5):e0127260. doi: 10.1371/journal.pone.0127260 (PMC4439077; doi:10.1371/journal.pone.0127260)
Supplement: S2 Table — (DOCX) [file pone.0127260.s012.docx]

| S2 Table: Extreme values of NDD when initial mean values were the same but differ in the initial range. | | | | | | | | | |
| --- | --- | --- | --- | --- | --- | --- | --- | --- | --- |
|  | MINIMUM | | | |  | MAXIMUM | | | |
| Nr of conspecific neighbours | 1 or 2 | 3 or 4 | 5 or 6 | 7 or 8 |  | 1 or 2 | 3 or 4 | 5 or 6 | 7 or 8 |
|  | -0.7000 | -0.8000 | -0.9000 | -1.0000 |  | -0.1000 | -0.3000 | -0.5000 | -0.7000 |
|  | -0.6625 | -0.7688 | -0.8750 | -0.9812 |  | -0.1375 | -0.3312 | -0.5250 | -0.7188 |
|  | -0.6250 | -0.7375 | -0.8500 | -0.9625 |  | -0.1750 | -0.3625 | -0.5500 | -0.7375 |
|  | -0.5875 | -0.7062 | -0.8250 | -0.9438 |  | -0.2125 | -0.3938 | -0.5750 | -0.7562 |
|  | -0.5500 | -0.6750 | -0.8000 | -0.9250 |  | -0.2500 | -0.4250 | -0.6000 | -0.7750 |
|  | -0.5125 | -0.6438 | -0.7750 | -0.9062 |  | -0.2875 | -0.4562 | -0.6250 | -0.7938 |
|  | -0.4750 | -0.6125 | -0.7500 | -0.8875 |  | -0.3250 | -0.4875 | -0.6500 | -0.8125 |
|  | -0.4375 | -0.5812 | -0.7250 | -0.8688 |  | -0.3625 | -0.5188 | -0.6750 | -0.8312 |
|  | -0.4188 | -0.5656 | -0.7125 | -0.8594 |  | -0.3812 | -0.5344 | -0.6875 | -0.8406 |
|  | -0.4000 | -0.5500 | -0.7000 | -0.8500 |  | -0.4000 | -0.5500 | -0.7000 | -0.8500 |
