# Supplementary material for: Lower Within-Community Variance of Negative Density Dependence Increases Forest Diversity
Source: PLoS One. 2015 May 20;10(5):e0127260. doi: 10.1371/journal.pone.0127260 (PMC4439077; doi:10.1371/journal.pone.0127260)
Supplement: S3 Table — (DOCX) [file pone.0127260.s013.docx]

S3 Table: Parameters used for supplementary simulations.

|  | Simulation | | | | | | | |
| --- | --- | --- | --- | --- | --- | --- | --- | --- |
|  | a | b | c | d | e* | f | g | h |
| Parameter |  |  |  |  |  |  |  |  |
| Grid size | 50X50 | 50X50 | 50X50 | 100X100 | 100X100 | 100X100 | 100X100 | 100X100 |
| Number of species | 25 | 50 | 100 | 25 | 50 | 100 | 50 | 50 |
| Seed production rate | 100 | 500 | 1000 | 100 | 500 | 1000 | 100 | 1000 |
| Adult mortality rate | 0.05 | 0.1 | 0.2 | 0.05 | 0.1 | 0.2 | 0.2 | 0.05 |
| Total seed dispersal distance | 2 | 5 | 8 | 2 | 5 | 8 | 5 | 5 |
|  |  |  |  |  |  |  |  |  |

* In this table, the set of parameters “e” are the ones used for simulations described in the main text.
