# Supplementary material for: Lower Within-Community Variance of Negative Density Dependence Increases Forest Diversity
Source: PLoS One. 2015 May 20;10(5):e0127260. doi: 10.1371/journal.pone.0127260 (PMC4439077; doi:10.1371/journal.pone.0127260)
Supplement: S4 Table — (DOCX) [file pone.0127260.s014.docx]

S4 Table: Regression statistics of the relationship between Shannon-index and initial mean values of NDD for different sets of parameters.

|  |  | Simulation | | | | | | | | |
| --- | --- | --- | --- | --- | --- | --- | --- | --- | --- | --- |
|  |  | a* | b | c | d | | e | f | g | h |
| Results ANOVA (regression) | R^2^ | 0.68 | 0.95 | 0.93 | | 0.64 | 0.95 | 0.96 | 0.93 | 0.98 |
|  | Regression coefficient | -0.57 | -1.70 | -2.27 | | -0.36 | -1.21 | -2.21 | -1.40 | -1.17 |
|  | p-value | 6.15E-03 | 5.96E-06 | 2.43E-05 | | 9.92E-03 | 9.60E-06 | 2.38E-06 | 2.96E-05 | 5.06E-07 |

* In this table, a, b,…h, correspond to the set of parameters described in Table S3.
