# Supplementary material for: Lower Within-Community Variance of Negative Density Dependence Increases Forest Diversity
Source: PLoS One. 2015 May 20;10(5):e0127260. doi: 10.1371/journal.pone.0127260 (PMC4439077; doi:10.1371/journal.pone.0127260)
Supplement: S5 Table — (DOCX) [file pone.0127260.s015.docx]

S5 Table: Regression statistics of the relationship between Shannon-index and initial range of NDD strength for different sets of parameters.

|  |  | Simulation | | | | | | | | |
| --- | --- | --- | --- | --- | --- | --- | --- | --- | --- | --- |
|  |  | a* | b | c | d | | e | f | g | h |
| Results ANOVA (regression) | R^2^ | 0.93 | 0.91 | 0.95 | | 0.94 | 0.98 | 0.98 | 0.98 | 0.96 |
|  | Regression coefficient | 2.48 | 1.75 | 1.53 | | 3.05 | 2.98 | 2.23 | 2.60 | 3.37 |
|  | p-value | 3.04E-05 | 6.15E-05 | 7.84E-06 | | 1.19E-05 | 2.04E-07 | 6.18E-07 | 1.58E-07 | 4.22E-06 |

* In this table, a, b,…h, correspond to the set of parameters described in Table S3.
